# Serum BDNF levels and state anxiety are associated with somatic symptoms in patients with panic disorder

**DOI:** 10.3389/fpsyt.2023.1168771

**Published:** 2023-07-18

**Authors:** Jiaxin Li, Ru Li, Dazhi Li, Jian Zhang, Xingguang Luo, Yong Zhang

**Affiliations:** ^1^Unit of Bipolar Disorder, Tianjin Anding Hospital, Tianjin, China; ^2^Department of Psychiatry, Yale University School of Medicine, New Haven, CT, United States

**Keywords:** brain-derived neurotrophic factor, escitalopram, panic disorder, somatic symptoms, state anxiety

## Abstract

**Background:**

We aimed to explore the predictive role of serum BDNF and anxiety-related variables in changes in somatic symptoms post-escitalopram treatment in panic disorder (PD) patients.

**Methods:**

Ninety PD patients and 99 healthy controls (HCs) were enrolled. PD patients received an 8-week escitalopram treatment. All patients were administered the Panic Disorder Severity Scale–Chinese Version (PDSS-CV) and State-Trait Anxiety Inventory (STAI) to assess panic and anxiety-related symptoms, respectively. Patient Health Questionnaire 15-item scale (PHQ-15) was performed to measure somatic symptoms, and the blood sample was collected to detect serum BDNF levels in all participants. We performed partial correlation analysis and multiple linear regression to explore correlates of PHQ-15 and predictors of PHQ-15 changes post-escitalopram treatment after controlling for age, gender, education levels (set as a dummy variable), the current duration, comorbid AP, and/or GAD.

**Results:**

Compared to HCs, PD patients had lower serum BDNF levels and higher PHQ-15 scores that could be improved post-escitalopram treatment. Lower baseline STAI state (*b* = −0.07, *p* = 0.004), and PDSS-CV scores (*b* = −0.25, *p* = 0.007), but higher baseline serum BDNF levels (*b* = 0.35, *p* = 0.007) contributed to the prediction of PHQ-15 changes post-escitalopram treatment.

**Conclusion:**

State anxiety, serum BDNF levels, and panic severity could predict changes in somatic symptoms post-escitalopram treatment, our results highlighted that serum BDNF could serve as a biological indicator for improving somatic symptoms in PD patients.

## 1. Introduction

Panic disorder (PD) is a subtype of anxiety disorders (ADs), characterized by recurrent and unexpected panic attacks (PAs) accompanied by somatic symptoms and persistent avoidance behavior (1), with a lifetime prevalence of 1.7% cross-national (2), and 3.4% in China (3), respectively. PD patients may have somatic symptoms as the core symptom, manifesting as headaches, palpitations, chest pain, fainting spell, and other non-specific symptoms commonly seen in clinical practice, resulting in misdiagnosis, and increased medical utilization (1). Somatic features related to anxiety have existed across the whole duration of MDD (4), while somatic features could be potentially influenced by some confounding factors such as age, gender, and education levels (5). The pathophysiology of PD remains unclear, some reviews supported that immune inflammatory factors, neuroimaging, and neuropeptide may contribute to panic disorder (6–8). However, little is known about the diagnostic or prognostic indicators of somatic symptoms in PD (9).

Brain-derived neurotrophic factor (BDNF) played a vital role in neuronal growth, differentiation, survival, and synaptic plasticity (10), which was associated with the pathogenesis of various psychiatric diseases (11). Its diagnostic value in Generalized Anxiety Disorder (GAD) (12), Obsessive-Compulsive Disorder (OCD) (13), and Major Depressive Disorder (MDD) (14) has been verified. Our previous findings have evidenced the role of serum BDNF played in the prediction and discriminant value using healthy controls and PD samples (15). Increasing evidence has pointed out that lower serum BDNF level was involved in PD (16) and poor treatment response (17), and a meta-analysis verified that antidepressants can exert an anti-panic effect through the promotion of BDNF expression (18). Christensen et al. (19) evidenced that psychosomatic features regarded as core symptoms could be reduced by antidepressant treatment. Additionally, changes in somatic symptoms could be associated with antidepressant treatment in MDD, ADs, and other psychiatric diseases (9). MDD patients with lower BDNF concentrations were more likely to suffer frequent somatic complaints (20), and remitted patients with MDD under Selective Serotonin Reuptake Inhibitors (SSRIs) treatment had less severe somatic symptoms compared to non-remitters (21), that is, the efficacy of SSRIs can predict somatic symptoms severity at the endpoint. Escitalopram has been shown to have a higher remission rate and a low risk of adverse events in PD treatment (22). Hitherto, none of the studies focused on the correlation between BDNF and somatic symptoms in PD, nor on predicting changes in somatic symptoms post-escitalopram treatment.

State anxiety means a condition- or situation-specific anxiety typically manifested as physiological arousal and consciously perceived feelings of worry, fear, and nervousness (23). PD patients were accompanied by autonomic arousal during PAs and were more sensitive to bodily changes than GAD patients (24). Higher alexithymia level was not only associated with dominant state/trait anxiety but also with panic severity and aggravated somatic symptoms in PD patients (25). Goto et al. (26) found that psychiatric patients who reported unexplained dizziness scored higher in the State-Trait Anxiety Inventory (STAI) state/trait. Thus, it is tentatively inferred that there is an association between state/trait anxiety and somatic symptoms in ADs. Till now, only a clinical trial focused on the association between state/trait anxiety and swallowing difficulty in PD patients (27), but this feature could not represent core somatic symptoms in PD. In all, studies are scarce on the correlation between state/trait anxiety and PD patients’ somatic symptoms.

As far as we know, there were no data combining serum BDNF and anxiety-related variables to explore the correlates of somatic symptoms in patients with panic disorder. Thus, we hypothesized that serum BDNF and state/trait anxiety were valuable as predictive indicators for changes in somatic symptoms in PD patients. The aims of our study are to: (1) discuss the correlation between serum BDNF, STAI, Panic Disorder Severity Scale–Chinese Version (PDSS-CV), and Patient Health Questionnaire 15-item scale (PHQ-15) at baseline and; (2) explore the predictive value of serum BDNF, state/trait anxiety, and panic-related symptom *per se* on the changes in somatic symptoms post-escitalopram treatment in PD patients.

## 2. Materials and methods

### 2.1. Participants

Panic disorder patients aged 18 to 65 years were recruited from outpatient or inpatient departments in Anding Hospital and Chest Hospital in Tianjin, China. PD patients were enrolled if they had: (a) a diagnosis of PD according to the Diagnostic and Statistical Manual of Mental Disorders, Fifth Edition (DSM-5); (b) a minimum score of 10 on the Panic Disorder Severity Scale–Chinese Version (PDSS-CV). Besides, PD patients were excluded if they: (a) had severe physical and (or) mental diseases including schizophrenia, bipolar disorder, mental retardation, etc., alcohol or substance abuse; (b) mood disorder such as bipolar disorder and MDD that could receive any antidepressant therapy. Healthy controls (HCs) matched in age, gender, and education levels were recruited from the local community. The study was conducted according to the Declaration of Helsinki. This study protocol was approved by the Ethics Committees of Tianjin Anding Hospital (NO: 2015-09), and written informed consent was gained from all participants.

### 2.2. Design

This was a case-controlled and prospective study to assess the correlation between serum BDNF levels, state/trait anxiety, and panic-related symptoms *per se* with somatic symptoms in PD patients, further exploring the possible prediction of changes in somatic symptoms post-intervention. To be specific, they were treated only by escitalopram, with a 10 mg/day fixed dose during the following 8 weeks. After enrollment, both PD patients and HCs accepted the sociodemographic characteristics, somatic symptoms assessments, and blood sample collection. Additionally, in PD patients, clinical characteristics including PDSS-CV, PHQ-15, and STAI were assessed while serum BDNF detection was performed. Meanwhile, sedative-hypnotic medications were allowed to have short-term use to relieve sleeplessness, including zolpidem (5–10 mg/day) and zopiclone (7.5 mg/day). Yet, benzodiazepines such as clonazepam and alprazolam could not be used to improve insomnia due to the characteristics of cognitive impairment.

### 2.3. Measurements

#### 2.3.1. Sociodemographic information and clinical assessments

A self-designed questionnaire was used to collect general sociodemographic information, including age, gender, marriage, education levels, occupation status, first-episode/relapse, family history, duration of illness, and the current duration.

Panic Disorder Severity Scale–Chinese Version (28) is a brief and standardized instrument, widely used to evaluate the severity of panic disorder with an established diagnosis and to monitor the treatment response in clinical work and scientific research, which includes seven domains rated on a 5-point scale from 0 to 4, with a higher score indicating more severe panic symptoms.

State-Trait Anxiety Inventory (23) is used to measure anxiety symptoms and general anxiety tendencies, and it consists of two subscales- STAI state (i.e., state anxiety) and STAI trait (i.e., trait anxiety), each with 20 items ranging from 1 to 4. STAI state has assessed the current state of anxiety, while STAI trait has assessed relatively stable aspects of “anxiety proneness.”

Patient Health Questionnaire 15-item scale (29) is intended to function as a self-administered instrument of somatic symptom severity, which has good performance characteristics. The patients were asked to rate the severity of their symptoms in the past month on a 3-point scale from 0 to 2 and summated scores of 0–4, 5–9, 10–14, and 15–30 indicating minimal, mild, moderate, and severe somatic symptoms, respectively.

In PD patients, PDSS-CV and PHQ-15 were evaluated both at baseline and week 8, while the STAI was solely assessed at baseline. Meanwhile, we set PHQ-15 changes from baseline to week 8 as the dependent variable, and prediction for PHQ-15 changes was observed post-escitalopram treatment.

All scale scores were assessed by two professional psychiatrists who had received a clinical training program, with a good level of inter-rater reliability, i.e., interclass correlation coefficients were above 0.80.

#### 2.3.2. Serum brain-derived neurotrophic factor (BDNF) levels

Antecubital venous blood samples (5 mL) were drawn from both PD and HC individuals at baseline, while blood samples of PD patients were collected again at week 8 to detect serum BDNF concentrations (ng/ml), which were collected into anticoagulant-free EDTA tubes between 7 and 8 AM and processed within 1 h of collection to reduce denaturation. After 1 h of incubation, serum samples were separated by centrifugation and stored at −80°C for further analyses. Serum BDNF levels were measured by enzyme-linked immunosorbent assay kits, according to the standard procedures for the kit (DG10522H, Lvyuan Biotechnology), and the optical density of the color reaction was read at 450 nm within 15 min utilizing a standard microplate reader (EL10A; BIOBASE).

### 2.4. Statistical analysis

Sociodemographic information, clinical-related variables (PDSS-CV, STAI, and PHQ-15), and serum BDNF levels between the PD group and HC group, or between the mild to moderate somatic symptoms group and severe somatic symptoms group were compared by utilizing the analyses of independent sample *t*-tests for continuous, and chi-square tests for categorical variables. Paired tests were used to assess the significance of changes in within-group continuous variables from pre- to post-treatment. Age, gender, education levels (set as a dummy variable), the current duration, comorbid AP, and/or GAD were included as covariates in the correlation and regression analyses. Partial correlation analysis was used to explore the correlation between PHQ-15, serum BDNF, and anxiety-related variables (PDSS-CV and STAI). Multiple linear regression analysis was used to clarify the predictive indicators for the PHQ-15 changes. Preliminary analyses were performed to render certain no violation of the assumptions of normality, linearity, multicollinearity, and homoscedasticity. Continuous variables were presented as mean (standard deviation) while categorical variables were presented as frequency (percentage).

All statistical analyses were calculated utilizing SPSS version 26.0, and besides, Gpower 3.1 was used to calculate the sample size and verify the statistical power, GraphPad Prism version 9.0 was used to generate graphs. The level of statistical significance was set *p*-value was 0.05 or less for two-tailed.

## 3. Results

### 3.1. Sociodemographic and clinical characteristics, as well as serum BDNF levels between PD, and HC groups

According to Gpower 3.1, we needed at least 64 PD patients and 64 HCs, and the actual power was 0.80. Actually, a total of 116 PD patients and 102 HCs were recruited in our study. Twenty-six PD patients dropped out because of incomplete assessments during follow-up, while three HCs due to incomplete blood sample collection. Finally, a total of 90 PD patients and 99 HCs completed the entire assessment and blood sample detection. We did not find differences in sociodemographic information, clinical-related variables, and serum BDNF levels between 26 dropped patients and 90 enrolled patients (all *p* > 0.05). There were no significant differences between the PD group and HC group in sociodemographic information, including age, gender, marriage, education levels, and occupation status (all *p* > 0.05). In addition, sixteen (17.8%) PD patients had a family history, and twenty-five (27.8%) were first-episode patients. For PD patients, we investigated the concomitant features of panic disorder with and without agoraphobia or GAD according to the PDSS-CV assessment, besides, we investigated the past presence of agoraphobia or GAD, and our findings showed that thirty-one (34.4%) of PD patients had concomitant features of agoraphobia (PDA), while thirty-eight (42.2%) of PD patients had concomitant features of GAD in the past.

Notably, our results revealed that PD patients showed lower serum BDNF levels (12.78 ± 2.40 vs. 22.41 ± 8.26 ng/ml, *p* < 0.001) and higher PHQ-15 scores (13.59 ± 4.74 vs. 2.98 ± 1.84, *p* < 0.001) than HCs. Besides, both female and male PD patients had strikingly lower serum BDNF levels (13.17 ± 2.47 vs. 21.76 ± 8.45 ng/ml; 12.18 ± 2.18 vs. 23.30 ± 8.00 ng/ml, all *p* < 0.001), and higher PHQ-15 scores (13.75 ± 4.78 vs. 3.02 ± 1.93; 13.34 ± 4.73 vs. 2.93 ± 1.73, all *p* < 0.001) compared to healthy females and males. In line with this, within the PD group, no gender differences in serum BDNF levels, PHQ-15, PDSS-CV, and STAI state/trait scores were found, either (all *p* > 0.05). Furthermore, patients with PDA showed higher PDSS-CV (17.10 ± 4.35 vs. 14.66 ± 3.12, *p* = 0.003) and STAI trait scores (58.03 ± 9.76 vs. 52.02 ± 11.59, *p* = 0.016) but did not differ in serum BDNF levels, PHQ-15, and STAI state scores compared to those without (all *p* > 0.05). Likewise, PD with versus without GAD groups showed no statistical differences in clinical-related variables and serum BDNF levels (all *p* > 0.05), all participants’ characteristics were summarized in Table 1.

**TABLE 1:** Sociodemographic characteristics, clinical scales scores, and serum BDNF levels between PD and HC groups.

Variables	PD (*n* = 90)	HCs (*n* = 99)	*t*/χ^2^	*P*
Age (years)^†^	47.62 (10.56)	49.95 (10.43)	-1.52	0.129
Gender (Male/Female)^#^	35/55 (38.9/61.1)	42/57 (42.4/57.6)	0.24	0.621
Marriage (yes/no)^#^	81/9 (90.0/10.0)	82/17 (82.8/17.2)	2.04	0.153
Education (L^a^/M^b^/H^c^)^#^	14/38/38 (15.6/42.2/42.2)	20/52/27 (20.2/52.5/27.3)	4.68	0.096
Occupation (laborer/staff)^#^	40/50 (44.4/55.6)	43/56 (43.4/56.6)	0.02	0.889
First-episode^#^	25/65 (27.8/72.2)	–	–	–
Family history^#^	16 (17.8)	–	–	–
PD with AP^#^	31 (34.4)	–	–	–
PD with GAD^#^	38 (42.2)	–	–	–
PDSS-CV^†^	15.50 (3.75)	–	–	–
STAI trait^†^	54.09 (11.31)	–	–	–
STAI state^†^	53.72 (12.98)	–	–	–
PHQ-15^†^	13.59 (4.74)	2.98 (1.84)	19.92	<0.001
BDNF^†^ (ng/ml)	12.78 (2.40)	22.41 (8.26)	-11.09	<0.001

PD, panic disorder; HCs, healthy controls; ^a^, primary school; ^b^, middle and high school; ^c^, college and above; AP, agoraphobia; GAD, Generalized Anxiety Disorder; PDSS-CV, Panic Disorder Severity Scale–Chinese Version; STAI, State-Trait Anxiety Inventory; PHQ-15, Patient Health Questionnaire 15-item scale; BDNF, brain-derived neurotrophic factor; ^†^, Mean (SD), *p*-value corresponds to independent samples *t*-tests; ^#^, n (%), *p*-value corresponds to chi-square tests.

**FIGURE 1:**
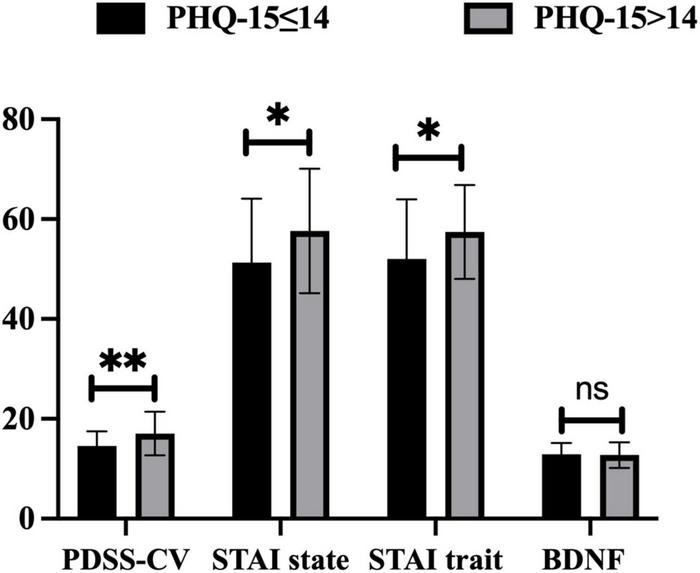
Differences in PDSS-CV, STAI state, STAI trait scores, and serum BDNF levels between PHQ-15 ≤ 14 and PHQ-15 > 14 group. PHQ-15, Patient Health Questionnaire 15-item scale; PDSS-CV, Panic Disorder Severity Scale–Chinese Version; STAI, State-Trait Anxiety Inventory; BDNF, brain-derived neurotrophic factor; *: *p* < 0.05, **: *p* < 0.01, ns: *p* > 0.05.

According to Kroenke’s theory (29), we divided PD patients into the mild to moderate somatic symptoms (PHQ-15 ≤ 14) group and the severe somatic symptoms (PHQ-15 > 14) group using a cut-off value of 14. Compared to the mild to moderate somatic symptoms group, PD patients with severe somatic symptoms showed higher scores of PDSS-CV (17.06 ± 4.37 vs. 14.51 ± 2.94, *t* = −3.04, *p* = 0.004), STAI state (57.66 ± 12.46 vs. 51.22 ± 12.79, *t* = −2.35, *p* = 0.021), and STAI trait (57.43 ± 9.40 vs. 51.96 ± 11.97, *t* = −2.29, *p* = 0.025), but no differences in serum BDNF levels (12.74 ± 2.57 vs. 12.81 ± 2.31, *t* = 0.14, *p* > 0.05) by performing paired *t*-tests, see Figure 1.

### 3.2. Improvements in clinical characteristics and serum BDNF levels pre- to post-escitalopram treatment in PD group

**FIGURE 2:**
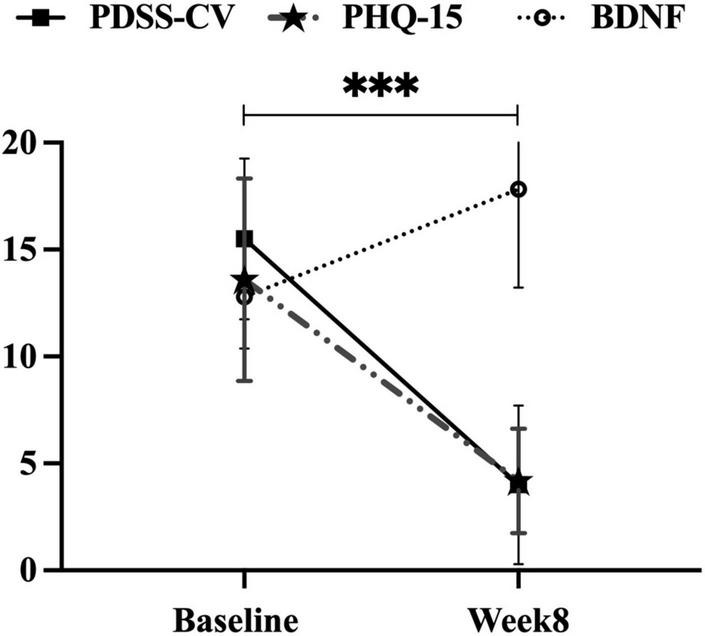
Improvements in PDSS-CV, PHQ-15 scores, and serum BDNF levels in the PD group. PDSS-CV, Panic Disorder Severity Scale–Chinese Version; PHQ-15, Patient Health Questionnaire 15-item scale; BDNF, brain-derived neurotrophic factor; ***, *p* < 0.001, the first two variables were subjected to paired *t*-test and the latter variable to paired rank sum test.

After 8 weeks of escitalopram treatment, significantly reduced scores were observed in PDSS-CV (15.50 ± 3.75 vs. 4.48 ± 3.71, *t* = 29.27, *p* < 0.001) and PHQ-15 (13.59 ± 4.74 vs. 4.18 ± 2.44, *t* = 27.59, *p* < 0.001) by performing paired *t*-tests, and significantly increased serum BDNF levels were found by utilizing paired rank-sum test (12.78 ± 2.40 vs. 17.82 ± 4.59 ng/ml, *Z* = −7.76, *p* < 0.001), see Figure 2.

### 3.3. Correlation analysis of baseline PHQ-15 in PD group

**FIGURE 3:**
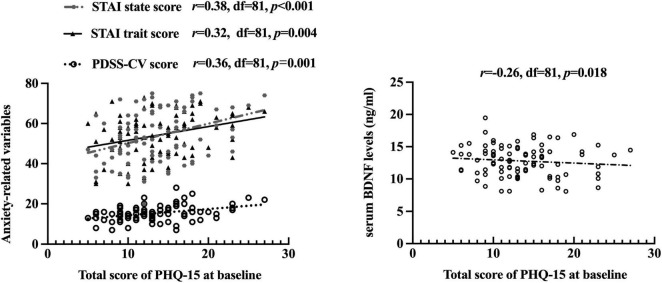
Correlation between PHQ-15 scores, other clinical scales scores, and serum BDNF levels in the PD group. The partial correlation was performed after controlling for age, gender, education levels (set as a dummy variable), the current duration, comorbid AP, and/or GAD; PHQ-15, Patient Health Questionnaire 15-item scale; STAI, State-Trait Anxiety Inventory; PDSS-CV, Panic Disorder Severity Scale–Chinese Version; BDNF, brain-derived neurotrophic factor.

After controlling for age, gender, education levels (set as a dummy variable), the current duration, comorbid AP, and/or GAD, partial correlation analysis was performed to clarify the correlation between serum BDNF, anxiety-related variables, and PHQ-15 in PD group. Higher baseline PDSS-CV (*r* = 0.36, *p* = 0.001), STAI state (*r* = 0.38, *p* < 0.001), and STAI trait (*r* = 0.32, *p* = 0.004) scores, but lower serum BDNF levels (*r* = −0.26, *p* = 0.018) were associated with higher baseline PHQ-15 total scores. Besides, STAI state scores (*r* = 0.32, *p* = 0.003) were positively correlated with PDSS-CV scores, see Figure 3. Additionally, lower baseline PDSS-CV (*r* = −0.38, *p* < 0.001), STAI state (*r* = −0.38, *p* < 0.001), and STAI trait (*r* = −0.29, *p* = 0.009) scores, but higher serum BDNF levels (*r* = 0.26, *p* = 0.018) were associated with greater PHQ-15 changes post-escitalopram treatment. However, at the end of week 8, we did not find a correlation between PHQ-15, PDSS-CV and serum BDNF, nor did we find a correlation between changes in these variables (all *p* > 0.05).

### 3.4. Regression analysis of PHQ-15 changes in PD group

Using multiple linear regression analysis, we analyzed predictive indicators of changes in somatic symptoms in PD patients. We chose the PHQ-15 changes as the dependent variable to explore the predictive roles of baseline independent variables, i.e., serum BDNF levels and anxiety-related variables. Even controlling for age, gender, and education levels (set as a dummy variable), the current duration, comorbid AP, and/or GAD, our results revealed that baseline STAI state scores (*b* = −0.07, *p* = 0.004), serum BDNF levels (*b* = 0.35, *p* = 0.007), and PDSS-CV scores (*b* = −0.25, *p* = 0.007) contributed to the prediction of PHQ-15 changes, i.e., changes in somatic symptoms post-escitalopram treatment, and the explanation of these three variables for the regression model was 31.3% (*F* = 5.05, *p* < 0.001), see Table 2.

**TABLE 2:** Multiple linear regression analysis of changes in somatic symptoms in PD patients.

Coefficient^a^									
**Variables**	**Unstandardized**		**Standardized**	***t***	***P***	**95% *CI***		**VIF**	***F***
	***B***	***SE***	**β**			**Lower**	**Upper**		
Constant	-5.712	3.474	–	-1.644	0.104	-12.626	1.202	–	5.05***
STAI state	-0.072	0.024	-0.290	-3.000	0.004	-0.120	-0.024	1.21	
PDSS-CV	-0.246	0.089	-0.286	-2.768	0.007	-0.423	-0.069	1.38	
BDNF	0.348	0.125	0.258	2.773	0.007	0.098	0.597	1.12	

^a^, the dependent variable was controlled for age, gender, education levels (set as a dummy variable), the current duration, comorbid AP, and/or GAD; PDSS-CV, Panic Disorder Severity Scale–Chinese Version; STAI, State-Trait Anxiety Inventory; BDNF, brain-derived neurotrophic factor; ***, *p* < 0.001.

## 4. Discussion

This was the first case-controlled and prospective study to explore the predictive roles of serum BDNF and anxiety-related variables in changes in somatic symptoms measured by PHQ-15 in patients with PD. The main findings in our study included: (1) Compared to HCs, PD patients had lower serum BDNF levels and higher PHQ-15 scores that could be improved after 8 weeks’ escitalopram treatment; (2) PD patients with higher baseline STAI state, STAI trait, and PDSS-CV scores, but lower serum BDNF levels could present severe somatic symptoms; (3) State anxiety, panic severity *per se*, and serum BDNF levels contributed to the prediction of PHQ-15 changes post-escitalopram treatment in PD patients. Consistent with our hypothesis, our results highlighted higher levels of serum BDNF predict greater changes in somatic symptoms post-escitalopram treatment in PD patients.

### 4.1. Somatic symptoms in PD patients

In line with previous studies, our findings supported that PD patients presented severe panic and somatic symptoms that could be improved post-escitalopram treatment (30, 31). Similarly, several studies have shown that antidepressants can alleviate somatic symptoms of MDD, ADs, and other psychiatric diseases (9, 19, 21). The previous review has considered an abnormal 5-HT function may involve the development and modulation of somatic symptoms (9), to which we added to evidence that the improvement of somatic symptoms paralleled the improvement of panic symptoms, possibly due to the involvement of a similar serotonin mechanism. In another aspect, Chinese patients paid more attention to somatic symptoms instead of emotional expression (25), possibly aggravating the severity of somatization. Besides, abnormal function of the pre-, post-, and para-central gyrus may play a vital role in the pathophysiology of somatic symptoms in MDD (32), indicating the neuroimaging mechanism of somatic symptoms. Given the similar pathophysiology (33), future imaging of PD patients with severe somatic symptoms could be performed. When compared to patients with PDA, there were discernible differences in panic symptoms, but not somatic symptoms in PD patients. Consistent with a previous study that revealed agoraphobia should be characterized as a severe subtype of panic disorder, and the presence of agoraphobia may be a proxy for panic severity (34). Likewise, most PD patients may present various GAD presence in the past time. Some authors even reported that GAD and panic attacks were lumped together under the same illness (35). That is to say, it is hard to differentiate simply PD from comorbid agoraphobia or GAD when assessment and diagnosis could be completed.

### 4.2. Association between serum BDNF levels and somatic symptoms in PD patients

Consistent with the previous study in GAD, and OCD (12, 13), we evidenced lowered serum BDNF levels again in PD patients (16). A review also verified lower BDNF both in MDD and bipolar disorder (11). But several studies concluded that there were no differences in BDNF concentrations between ADs and HCs (17, 36). The different results may be due to the source of BDNF (plasma-derived or serum-derived) and different subtypes of ADs could confound the homogeneity of the sample (37). Moreover, serum BDNF levels are negatively associated with PD patients’ somatic symptoms, and the strength of the current study showed that higher levels of serum BDNF predicted greater changes in somatic symptoms after 8 week’ escitalopram treatment. In line with the previous study that PD patients with lower serum BDNF levels were more likely to have a poor response to panic intervention (17). Another review pointed out that antidepressants could reverse decreased BDNF levels for MDD patients, indicating BDNF may serve as a promising predictor of treatment response (38). In all, BDNF might regulate neurogenesis to achieve anxiolytic/antidepressant-like effects (39). Interestingly, several studies have confirmed MDD patients with lower BDNF concentrations reported frequent somatic complaints (20), and somatic symptoms interacted with depressive symptoms (32), both can be improved by SSRIs treatment (21). Our findings concluded higher BDNF levels may contribute to the improvement of somatic symptoms in PD patients, supporting the notion that serum BDNF serving as a potential peripheral biomarker may predict response to antidepressant treatment. Namely, we speculated that BDNF may contribute to the change of the somatic features by improving anxiety symptoms, while somatic symptoms may be served as a core feature in anxiety disorder (40).

### 4.3. Association between panic-related symptoms *per se* and somatic symptoms in PD patients

Unsurprisingly, our results revealed a significant negative trend between changes in somatic symptoms and baseline panic severity. A meta-analysis (41) confirmed that the higher severity of panic-related symptoms at baseline was associated with poorer clinical improvement, our result was also replicated in an RCT study consisting of 232 PD patients (42). Likewise, in MDD individuals, higher scores of anxiety could contribute to apparent somatic symptoms (43), while depressive symptoms would also worsen somatic symptoms, and vice versa (32). Thus, we speculated that somatic symptoms were also relieved by improvement in panic severity post-SSRIs treatment. This is also the reason why there was a correlation between PHQ-15, PDSS-CV, and serum BDNF before treatment, but this correlation disappeared during and after treatment. This might be due to the combined effects of the antidepressant intervention itself, as well as improvement in panic symptoms, and an increase in serum BDNF levels post-intervention, which significantly improved the somatic symptoms.

### 4.4. Associations between state anxiety and somatic symptoms in PD patients

Our findings also displayed that both trait and state anxiety had a positive correlation with somatic symptom severity in PD patients even though controlling age, gender, education levels (set as a dummy variable), the current duration, comorbid AP, and/or GAD. Moreover, when compared to the mild to moderate somatic symptoms group, PD patients with severe somatic symptoms exhibited higher levels of STAI state and STAI trait. However, we only found state anxiety may predict changes in somatic symptoms post-escitalopram treatment. The previous study showed that state anxiety not only accounted for changes in functional somatic symptoms over time (44) but was also associated with avoidance behavior during PAs (45). A plausible explanation for this was that patients may exhibit somatic symptoms as a coping strategy when they suffered temporary unpleasant emotional arousal from PAs (25) to reduce state anxiety in condition- or situation-specific. Contrary to a previous finding in patients with functional gastrointestinal disorders (46), we did not find a predictive role of trait anxiety in somatic symptoms. We considered that state anxiety may be more likely to be related to acute anxiety, which was sensitive to respond to changes in somatic symptoms. However, trait anxiety is a relatively stable variable that tough to change over short-term intervention (47). Besides, Roberts et al. (48) found that the trait version of the Cognitive and Physical Anxiety State Characteristics Scale (STICSA), rather than the trait version of the STIA, was the better measure of somatic symptoms of anxiety, and the latter owing weaker correlation.

### 4.5. Limitations

Our study definitely had several limitations. First, PD patients with poorer overall functions showed more somatic complaints (49). Although we controlled age, gender, education levels, the current duration, comorbid AP, and/or GAD as confounders through multiple linear regression, we did not control for cognitive and severity of social functioning at baseline, which might influence clinical prognosis, so there might exist bias of selection in recruiting PD patients. Second, the short duration of the 8-week intervention may affect the prediction of changes in somatic symptoms. A review (50) pointed out that 10 weeks or more of SSRIs intervention contributed to improving PD patients’ clinical symptoms and overall functions, and we believe that the predictive efficacy may be better with a prolonged period of treatment. Third, although state anxiety, serum BDNF levels, and panic severity could predict changes in somatic symptoms in PD, these predictive factors only explained 31.3% of the regression model. Previous findings reported that alexithymia aggravated more somatic symptoms in PD (25), and MDD (43), indicating other influencing factors should be considered in further exploration. Fourth, a fixed dose of escitalopram (10 mg/day) controlled some confounding factors, but it could also delay the timing of treatment response. Besides, it was hard to analyze the differences among BDNF levels, panic symptoms, and somatic symptoms according to the dose of escitalopram. Therefore, our conclusions need further verification under a flexible dose of escitalopram. Last, although our study lack data on the STAI variable at week 8, it did not affect to discuss of whether baseline serum biomarker (serum BDNF) and anxiety-related variables (PDSS-CV and STAI) could predict the PHQ-15 changes post-escitalopram treatment. However, we need to explore the influence of changes of STAI on the improvement of somatic symptoms among PD patients in further study.

In summary, we performed the first case-controlled and prospective study combining serum BDNF and anxiety-related variables to explore predictive indicators of changes in somatic symptoms in PD patients. Our findings proposed that STAI state scores, serum BDNF levels, and PDSS-CV scores can prove useful in ensuring the target PD population, allowing for early therapeutic intervention, and predicting clinical prognosis. Future studies with larger sample sizes, long-term intervention, and flexible doses of escitalopram are needed to replicate our results and explore the predictive role of trait anxiety in PD patients’ somatic symptoms.

## 5. Conclusion

State anxiety, serum BDNF levels, and panic severity could predict changes in somatic symptoms post-escitalopram treatment, our results highlighted that serum BDNF could serve as a biological indicator for improving somatic symptoms in PD patients.

## Data availability statement

The raw data supporting the conclusions of this article will be made available by the authors, without undue reservation.

## Ethics statement

The studies involving human participants were reviewed and approved by the Ethics Committees of Tianjin Anding Hospital (NO: 2015-09). The patients/participants provided their written informed consent to participate in this study.

## Author contributions

JL and YZ designed the study and obtained the data. RL undertook the analysis supervised by YZ. JL and RL wrote the manuscript. DL, JZ, and XL performed the survey. All authors read the final manuscript and agreed with the text.
